# Supplementary material for: Characterization of Rhizosphere Microbial Diversity and Selection of Plant-Growth-Promoting Bacteria at the Flowering and Fruiting Stages of Rapeseed
Source: Plants (Basel). 2024 Jan 22;13(2):329. doi: 10.3390/plants13020329 (PMC10819753; doi:10.3390/plants13020329)
Supplement: Supplementary file 1 [file plants-13-00329-s001.zip › plants-2817469-supplementary.pdf]

Supplementary Figure S1

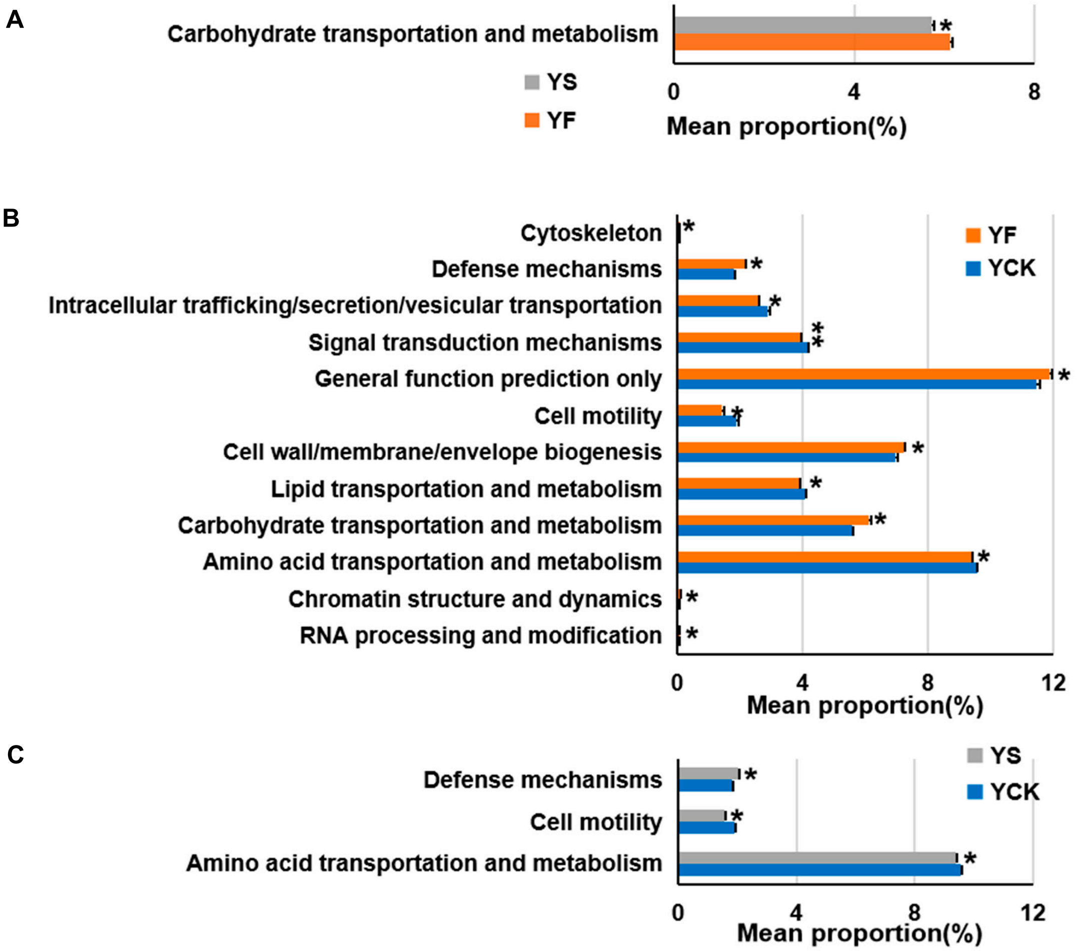

Supplementary Figure S1. The difference in the COG functional prediction between the YF, YS and YCK groups. (A) the difference in the COG abundance prediction between the YF and YS groups,(B) the difference in the COG abundance prediction between the YF and YCK groups,(C) the difference in the COG abundance prediction between the YS and YCK groups.*p<0.05; **p<0.01

Supplementary Table S1: Classifcations of identifed plant growth promoting rhizobacteria in different soil samples

Strain	Silicate bacteria mediums	SLP medium		
		Supplemented with 10 mg/L of Cu	Supplemented with 700 mg/L of Zn	Supplemented with 4000 mg/L of Pb
YF3	+	-	-	-
YF4	-	-	-	+
YF6	-	-	+	+
YF7	-	+	+	-
YF12	-	-	-	-
YF13	+	+	-	-
YF15	+	-	-	+
YF16	-	-	+	-
YF20	+	+	-	-
YF21	+	+	+	+
YF25	-	-	-	-
YF26	+	-	-	-
YF28	-	-	-	-
YF29	+	-	-	-
YF30	-	+	-	+
YF32	+	+	-	+
YF33	-	+	-	+
YF35	-	-	+	-
YF43	+	+	+	-
YF45	-	-	-	-
YF51	-	-	-	+
YF52	-	-	-	-
YF53	+	-	+	+
YF57	-	-	-	+
YF58	+	+	-	-
YF60	-	-	-	-
YF61	-	-	-	+
YF63	+	+	-	+
YF64	+	-	-	-
YF66	-	-	-	+
YF67	-	-	-	+
YF70	-	-	+	-
YF71	-	+	-	-
YF72	+	+	-	+
YS1	+	+	-	-
YS2	+	-	-	-
YS3	-	+	-	+
YS12	-	-	+	-
YS13	-	+	-	+
YS19	-	-	-	-
YS20	-	-	-	+
YS22	-	-	-	-
YS23	+	+	+	-
YS24	-	-	-	-
YS28	-	-	-	-

YS30	-	+	-	+
YS31	+	+	-	-
YS32	-	-	-	+
YS34	-	-	+	+
YS36	-	+	-	-
YS38	-	-	+	-
YS43	-	-	-	+
YS44	-	-	+	+
YS45	-	-	-	-
YS46	-	-	+	+
YS53	-	-	-	+
YS54	-	-	-	-
YS55	-	-	-	-
YS61	-	+	+	+
YS62	-	-	-	-
YS64	-	-	-	+
YS65	-	+	+	+
YS66	-	-	-	-
YS67	-	-	-	-
YS70	-	-	+	+

Supplementary Table S2:The rhizosphere soil microbial community in flowering and fruiting stages of rapeseed were significantly different from the control group

	Relative abundance of microbial community						
Bacterial phyla	Proteobacteria	Bacteroidetes	Chloroflexi	Patescibacteria	Planctomycetes	Nitrospirae	Rokubacteria
YCK	47.99%±3.33%a	9.43%±4.40%a	4.01%±2.33%a	2.10%±1.02%a	3.02%±0.70%a	1.68%±0.39%a	5.22%±1.78%
YF	24.04%±1.83%b	19.34%±3.81%b	12.71%±0.81%b	9.20%±1.47%b	4.87%±0.01%b	1.92%±0.80%a	-
YS	30.03%±3.36%c	17.36%±2.53%c	8.82%±1.02%c	3.42%±1.19%a	2.55%±0.84%a	3.08%±0.26%b	-
Bacterial genera	Ellin6067	Sphingomonas	Flavisolibacter	UTCFX1	ADurb.Bin063-1		
YCK	5.35%±1.76%a	1.02%±0.43%a	-	-	-		
YF	1.80%±0.52%b	2.90%±1.38%a	5.15%±0.65%a	6.20%±0.39%a	3.61%±0.35%a		
YS	1.11%±0.25%c	3.54%±1.33%b	2.40 %±1.24%a	2.68%±0.60%b	2.43%±0.26%b		
Fungal phyla	Mortierellomycota	Olpidiomycota	Ascomycota	Basidiomycota	Rozellomycota	Glomeromycota	
YCK	15.11%±0.68%a	-	31.96%±9.67%a	16.47%±4.28%a	-	10.78%±5.01%	
YF	78.61%±8.10%b	-	4.03%±0.84%b	3.39%±0.87%b	2.70%±1.23%a	-	
YS	21.93%±3.78%c	63.29%±1.96%	3.64%±2.28%bc	-	6.16%±3.90%a	-	
Fungal genera	Mortierella	Rhizophagus	Olpidium	Fusarium	Cystofilobasidium	Ceratobasidium	
YCK	1.51%±0.07%a	1.27%±0.19%	-	-	-	10.27%±2.65%	
YF	6.25%±0.16%b	-	-	3.78%±1.06%	2.20%±0.39%	-	
YS	1.66%±0.15%c	-	6.31%±0.16%	-	-	-	

Note: The '-' means the relative abundance of microbial community was lower than 1%.
The relative abundance was indicated by mean and standard deviation for triplicates. The data with different letter in each column was significantly different (P<0.05).
YF, YS and YCK mean rhizosphere soil samples collected from the roots of flowering stage of rapeseed, the roots of of fruiting stages, and the control check soil samples, respectively.
